# Shared Medication PLanning In Home Hospice to Address Medication Regimen Complexity and Family Caregiver Burden: A Brief Report

**DOI:** 10.1089/jpm.2024.0229

**Published:** 2024-10-28

**Authors:** Jennifer Tjia, Margaret F. Clayton, Geraldine Puerto, Vennesa Duodu, Francesca Troiani, Sruthi Tanikella, Susan DeSanto-Madeya

**Affiliations:** 1 Department of Population and Quantitative Health Sciences, UMass Chan Medical School, Worcester, Massachusetts, USA.; 2 College of Nursing, University of Utah, Salt Lake City, Utah, USA.; 3 College of Nursing, The University of Rhode Island, Kingston, Rhode Island, USA.

**Keywords:** burden, caregiver, decision-making, deprescribing, hospice, patient–clinician communication

## Abstract

**Objective:**

Medication management in home hospice is challenging for family caregivers (FCGs). We tested a patient-centered medication review and FCG support program delivered by hospice staff called “Shared Medication PLanning In (SiMPLIfy) Home Hospice.”

**Methods:**

A pilot cluster-randomized trial at two U.S. home hospice agencies measured the primary outcome of reduction in Medication Regimen Complexity Index (MRCI) (range 0 [no medications]–no upper limit) and secondary outcome of Family Caregiver Medication Administration Hassle Scale (FCMAHS) (range 0 [no hassle−120 [greatest hassle]).

**Results:**

Twenty-two patient-FCG dyads enrolled. Mean baseline MRCI in the intervention group = 39 (95% CI: 30.9, 47.1) and control group = 25.5 (95% CI: 21.0–30.1). Half of intervention patients (3 of 6) had reduced MRCI compared with 26.7% (4 of 15) control patients (*p* = 0.07). MRCI was not significantly associated with caregiver burden. FCMAHS differed between spousal and nonspousal FCGs (*p* = 0.12).

**Conclusion:**

A clinician-FCG-patient communication program in home hospice is feasible and may reduce medication complexity. SiMPLIfy has the potential to reduce polypharmacy.

## Introduction

Nearly 1.3 million Americans of age ≥65 years enroll in hospice annually; 45% receive care at home.^1^ They are prescribed 10–12 daily medications on average,^2–4^ yet have declining organ function that increases risk of drug-related harm.^5,6^ Hospice patients also take questionably beneficial medications^7–9^ while simultaneously being prescribed more and more symptom management medications.^4^ This changing combination of medications contributes to stress and confusion for family caregivers (FCGs) who are responsible for their administration.^10,11^

Growing evidence suggests that less may be more for medication prescribing in persons approaching end of life.^11–13^ A randomized controlled trial of statin discontinuation in hospice-eligible patients showed reduced medication burden and positive patient well-being.^13^ Translating such evidence into practice is challenging.

To address the challenge of hospice medication management, we developed a patient-centered, clinician-delivered, medication review, and FCG support program called Shared Medication PLanning In (SiMPLIfy) Home Hospice.^14^ This article reports preliminary results from a feasibility trial of SiMPLIfy’s impact on patient medication outcomes. We also report associations between baseline medication regimen complexity and family-reported medication administration hassle.

## Methods

### Study design and setting

We conducted this pilot cluster-randomized trial between October 2019 and March 2022 at two large, geographically distinct U.S. home hospice agencies. Within each hospice agency, two regional offices were selected by administrators and our research team as participating sites. This study was approved by the UMassChan Medical School institutional review board (IRB) (protocol #H00015417) and is registered on ClinicalTrials.gov NCT03972163.

### Study population

Patient inclusion criteria included being newly admitted to home hospice and of age ≥65 years old with advanced life-limiting illness; estimated life expectancy >1 month; recent functional status decline (i.e., Karnofsky Performance Status^15^ decline to <80% in prior 3 months); polypharmacy (i.e., ≥5 regularly scheduled medications [excluding antimicrobials]); cognitive ability to provide informed consent using a Short Portable Mental Status Questionnaire score ≥6^16^ OR a legally authorized representative able to provide proxy consent. FCGs were eligible if they: self-identified as “usually” or “always” providing care to the patient; were English-speaking; had telephone access and cognitive ability to participate. Family was operationally defined “any relative, partner, friend, or neighbor who has a significant personal relationship with the patient and provides a broad range of assistance.”^17^

### Randomization and intervention

Before trial initiation, sites within each hospice agency were randomly selected to be the intervention or control site. Patients/FCGs were blinded to allocation status. Intervention details are described elsewhere.^14^ Briefly, the intervention is a clinician-facing toolkit with an educational program that encourages hospice clinicians to reassess medication appropriateness as a patient’s illness progresses and to encourage FCG empowerment to manage medications. The intervention was developed by an interprofessional group of medical, nursing, and pharmacist experts to address challenges in the hospice setting. The SiMPLIfy program included a 1.5-hour training program that presented a standardized approach to goal-concordant medication review that emphasized ascertaining patient and FCG goals, as well as patients’ current medication use patterns (e.g., assessing passive nonadherence). Clinicians randomized to the intervention were provided with SiMPLIfy’s brief communication skills guides that included key phrases to use as deprescribing conversation starters. Control staff did not receive SiMPLIfy’s training in patient-centered medication review or communication, but did receive information about risks from polypharmacy and information from the National Institute of Aging’s [NIA] website on “Medicines and Medication Management.”^18^ All patient-FCG-facing booklets also included information from this NIA link,^18^ guidance about “What do I need to tell my Nurse or Doctor,” and a medication tracking worksheet. SiMPLIfy intervention booklets additionally contained information about “What is deprescribing?”. More information about the current version of the program is available upon request.

### Patient-FCG dyad recruitment

We worked with each hospice agency to refine case-finding methods consistent with IRB requirements while respecting hospice agency workflow. Informed consent was obtained over the phone; appropriate intervention (or control) study medication management workbooks were sent by express delivery to patients and FCG participants.

### Patient and FCG measures

The main outcome measure was the Medication Regimen Complexity Index (MRCI),^19–21^ which characterizes patients’ chronic disease, preventive, and symptom medication regimens. The MRCI score is the weighted sum of the dosage form, dosing frequency, and administration instructions.^20^ Minimum MRCI is 1.5 [a single tablet once a day], and maximum score increases with number of medications; 0 indicates no medication use. The secondary outcome was the FCG Medication Administration Burden, measured using the 24-item Family Caregiver Medication Administration Hassle Scale (FCMAHS); four subscales include Information Seeking/Sharing, Safety Issues, Scheduling Logistics, and Polypharmacy each rated from 0 (not a hassle) to 5 (one of the worst of all hassles) for a range of 0–120. Overall scale reliability is 0.95.^22^

### Data collection

Research assistants extracted hospice medical records for patient demographics and clinical data, including prescribed medications at baseline, 2, 4, 5, 8, 12, 16, 20, 24 weeks, and death. Research assistants conducted telephone assessments at those same intervals for the FCMAHS.

### Data analysis

Descriptive statistics characterize baseline patient and FCG characteristics, medication complexity, and FCG medication administration hassle. To estimate the preliminary intervention effect size, we compared the proportion of patients with MRCI declines between the intervention and control arms using a chi-square test. We explored the mean change between baseline and final MRCI by intervention status and examined the mean change between the baseline and final MRCI by intervention status using *t*-tests. Unadjusted linear regression examined the association between baseline MRCI and baseline Hassle Scale. One-way analysis of variance (ANOVA) characterized the association between baseline Hassle Scale score and the caregiver relationship to the patient. All analyses were conducted in Stata 17.0 (College Station, TX).

## Results

A total of 23 patient-FCG dyads enrolled; one patient died between enrollment and data collection. The final cohort of 22 patient-FCG dyads entered data collection. Among participating dyads, seven were recruited into the intervention arms, and 15 were recruited to the control arms. Recruitment yield was similar by arm, with 15% enrollment of 77 intervention patients eligible at time of eligibility determination (unenrolled included: 15 died before recruitment call, 23 passively refused, 22 actively refused) and 13.3% enrollment of 184 control patients eligible at time of eligibility determination (unenrolled included: 52 died before the recruitment call, 61 passively refused, 59 actively refused).

Of the 22 hospice patients, the mean age was 80.8 years, with the largest group comprised of those older than 90 years (31.8%; *n* = 7). Of the 22 patients, 86.4% (*n* = 19) were White and 95.5% (*n* = 21) identified as non-Hispanic. The majority of patients were female (68.2%; *n* = 15). The most common admitting diagnosis was cancer (50.0%), followed by cardiopulmonary disease (27.3%) and end-stage renal disease (18.2%). The mean baseline MRCI score among participants was 29.8 but differed by intervention status: intervention patients mean MRCI = 39 (standard error [SE] 3.3; 95% confidence interval [CI]: 30.9, 47.1) and control patients mean MRCI = 25.5 (SE 2.1; 95% CI: 21.0–30.1). There were 22 hospice caregivers, including 77.3% (*n* = 17) women. The dominant race among caregivers was White (81.8%; *n* = 18).

There was a wide range of polypharmacy upon referral to hospice (mean 13.0; standard deviation [SD] 4.4; range 6–23), admission to hospice (mean 20.7; SD 5.2; range 19–31), and at death (mean 25.7; SD 8.4; range 0–41). Six of the 22 participants had at least one of their original medications from the time of referral stopped during the hospice stay.

Of the seven intervention patients, six had an MRCI in follow-up, of whom three (50%) had a MRCI decrease during the trial. In contrast, four of 15 patients (26.7%) in the control arm had a MRCI score decrease between the beginning and end of enrollment. The *p* value for this difference is 0.07. The mean difference between baseline and last MRCI in the intervention arm was +4.2 (95% CI: −16.7, 25.0), and in the control arm was +10.1 (95% CI: 3.7, 16.5), *p* = 0.40. There were not enough FCMAHS between baseline and study completion to assess the intervention’s effect on FCMAHS scores.

In unadjusted linear regression, there was no significant association between MRCI and caregiver hassle (ß coef −0.13, *p* = 0.73). The mean spouse Hassle Scale score was 17.4 (SD 13.8), the mean for adult children was 39.1 (SD 19.1), and for other non-FCGs, it was 8.5 (SD 10.6). The *p* value between spousal and nonspousal caregivers’ hassle was 0.12 (*t*-test).

## Discussion

In this feasibility trial, conducted during the onset and height of the COVID pandemic, we found a higher proportion of reduced medication regimen complexity in patients randomized to the intervention compared with the control arm at *p* = 0.07, even though we were underpowered. Other than the statin discontinuation trial,^13^ there are no randomized deprescribing trials focused on hospice. Our pilot study provides proof of concept for our intervention to support goal-concordant prescribing in home hospice to red uce medication regimen complexity, which is feasible.

Our findings also show a difference in reported caregiver hassle depending on the caregiver’s relationship to the patient. This was an unanticipated but important finding. The difference in caregiver burden by relationship to the patient was mixed in a 2011 meta-analysis.^23^ In the setting of serious illness, findings are more consistent with our results. A cross-sectional study of caregivers of cancer patients reported that adult children experienced higher social/emotional caregiving burdens than spouses.^24^ Similar findings were reported in a large study of caregiving burden among dementia caregivers for patients prescribed cholinesterase inhibitors.^25^ Chappell et al. found greater reported burden among adult children than spousal caregivers; they also found that medication management was burdensome, was perceived as work, and increased over time.^25^

Challenges to deprescribing in hospice include patient and family fear of abandonment, fear that stopping medication will either hasten death or is a sign of imminent death, and lack of trust in the hospice clinician.^26^ At times, there may also be resentment that stopping medications in hospice is motivated by financial gain for the hospice rather than the best interests of the patient. We recommend an approach that emphasizes aligning medication prescribing with the goals of the patient, as well as having conversations about the unappreciated risk of harm from polypharmacy. Caregiver training can focus on explaining the benefit of simplifying medication regimens, as well as distinguishing between medications that are essential (e.g., for current symptom management) from nonessential medications (e.g., preventative for future complications or have little evidence of benefit). Helping individuals understand the rationale and benefit of deprescribing has been shown to ease concerns and facilitate medication changes.^27^

### Limitations

COVID affected recruiting and contributed to our small sample size, thus limiting generalizability. While there were intervention/control arm differences in patient characteristics and medication burden, we were unable to conduct multivariable analysis due to the limited power. Thus, results are not adjusted for patient demographics, diagnoses, severity of conditions, and differences in caregiver support. As such, any interpretation of these findings should be done with great care. Despite these limitations, we found signal of improvement in medication burden. This suggests promise for the intervention.

## Conclusion

SiMPLIfy is a novel approach to patient and FCG support for medication management, including deprescribing. Our pilot study provides proof of concept for our intervention to support goal-concordant prescribing in home hospice to reduce medication regimen complexity. Furthermore, research is needed to assess implementation and uptake in a larger population of hospices and to evaluate the effect in a fully powered trial.
